# The influence of a virtual reality entertainment program on depressive symptoms and sedentary behaviour in inpatient stroke survivors: a research protocol for a pilot randomized controlled trial

**DOI:** 10.1186/s40814-022-01189-8

**Published:** 2022-10-22

**Authors:** Isabelle Rash, Megan Helgason, Donna Jansons, Lindsay Mitchell, Brodie M. Sakakibara

**Affiliations:** 1 Graduate Program in Rehabilitation Sciences, The University of British Columbia, Vancouver, Canada; 2 Centre for Chronic Disease Prevention and Management, Southern Medical Program, The University of British Columbia Okanagan, 1088 Discovery Avenue, Kelowna, BC V1V 1V7 Canada; 3 Interior Health Authority, Kelowna, Canada; 4 Department of Occupational Science and Occupational Therapy, The University of British Columbia, Vancouver, Canada

**Keywords:** Post-stroke depression (PSD), Depressive symptoms, Sedentary behaviour, Virtual reality (VR)

## Abstract

**Background:**

Sedentary behaviour among stroke inpatients may be due to high rates of depressive symptoms after stroke. Thus, efforts to address depressive symptoms among stroke inpatients are warranted to in turn lessen sedentary behaviour. Despite evidence that virtual reality (VR) is emerging as a method to help with depression, the use of VR to improve depression among inpatient stroke survivors has yet to be studied. In this paper, we report on the protocol investigating the feasibility of a VR entertainment system at improving depressive symptoms among stroke survivors receiving inpatient rehabilitation.

**Methods:**

In this single-blind randomized controlled trial, 30 inpatient stroke survivors from the rehabilitation unit at Kelowna General Hospital will be randomized to either (1) intervention: 3 times per week of VR entertainment for duration of inpatient rehabilitation or (2) control: usual care. Individuals will be included if they have a confirmed diagnosis of stroke, are 19 years of age or older, able to provide informed consent, have physician clearance to participate in the study (medically stable or fit), or are able to understand English. Outcome measures to address depressive symptoms (primary outcome), sedentary behaviour, motivation, anxiety, stress, and happiness (secondary outcome) will be administered at two timepoints: (1) baseline (T1) and (2) post-intervention (T2). Study analyses will consider study feasibility indicators and clinical (statistical) outcomes. Means and standard deviations (for continuous variables) and frequencies and proportions (for categorical variables) will be used to summarize the variables. Feasibility indicators will be dichotomized into either ‘success’ if they meet the a priori criteria, or ‘revise’ if they do not meet the criteria. Intervention effects post-intervention (T2) for the primary and secondary clinical outcomes will be estimated using linear regression including baseline (T1) controlling for age and sex.

**Discussion:**

The results of this trial will add to our understanding of depression and sedentary behaviour among individuals receiving inpatient stroke rehabilitation as well as the feasibility of a VR entertainment program to improve depressive symptoms, which will in turn may lessen sedentary behaviour in inpatient stroke survivors.

**Trial registration:**

ClinicalTrials.gov Identifier: NCT04011202 . First posted July 8, 2019 (study postponed from March 2020 to July 2021 due to COVID-19).

**Supplementary Information:**

The online version contains supplementary material available at 10.1186/s40814-022-01189-8.

## Background

Stroke is a leading cause of morbidity and neurological disability worldwide [1]. After stroke, it is common for many individuals to experience ongoing functional, physical, and cognitive impairments, issues with the quality of social functioning, and deficits in sensory function, communication, and cognition [2]. Stroke sequelae in turn contribute to high levels and severity of post-stroke depression (PSD) and sedentarybehaviours [3, 4].

Depression and depressive symptoms after stroke are recurring occurrences with as many as 28% experiencing depressive disorders or symptoms one-month post-stroke, and 36% between 2 and 5 months [5]. During the first year after stroke, PSD occurs in approximately 33% of stroke survivors [6, 7]. Depressive symptoms among individuals receiving inpatient stroke rehabilitation are especially prevalent, with research showing rates as high as 78.3% in women and 65.2% in men [8].

Issues associated with depressive symptoms during inpatient rehabilitation are serious. Inpatient rehabilitation after stroke is a critical time for stroke survivors to optimize their recovery, regain independence and improve their quality of life [9–15]. Evidence indicates that people with PSD do not recover as well as those without PSD [16–20]. In a study assessing stroke survivors 3 and 15 months post-stroke, Pohjasvaara et al. [20] observed depression, especially major depression, as an independent predictor of poor long-term functional outcome of stroke. Furthermore, Schöttke et al. [16] reported that post-stroke depression during the acute phase after stroke is an important risk factor for long-term functional impairment and long-term depression.

Previous research has demonstrated a positive association between depressive symptoms and sedentary behaviour [21–23]. Excessive amounts of sedentary time are reported among stroke survivors, beginning in inpatient rehabilitation, and continuing post-discharge. The relationship between depressive symptoms and increased sedentary time is prominent, thus reducing depressive symptoms may enable increased activity [16]. Several studies have estimated that more than 75% of waking hours as an inpatient are sedentary [24–27], with estimates as high as 88% [26]. Furthermore, despite calls for highly intensive stroke rehabilitation, research indicates that the actual hours per week of therapy sessions is substantially lower than the 15 h per week recommended in best practice guidelines [28]. Compounding the lack of therapy is the amount of sedentary time during such sessions, with reports that 62% of physical therapy and 77% of occupational therapy are spent doing sedentary activities [24].

Following discharge from rehabilitation, rates of sedentary behaviour appear to mirror those reported as inpatients. Research suggests that stroke survivors living in the community spend approximately three quarters of their waking hours in sedentary behaviours, and engage in minimal walking following rehabilitation [3]. In fact, in a recent case–control study, it was shown that stroke survivors spend approximately 11 h per day sedentary, compared to 8 h per day for their age- and sex-matched counterparts [29]. High levels of moderate-intensity activity (60–75 min/day) may lower the increased risk of mortality associated with sedentary behaviours (> 8 h/day) [30]. However, achieving this level of activity may be difficult to reach among community-living stroke survivors due to environmental and health circumstances. Thus, interventions to reduce sedentary time as inpatients may be a promising therapeutic intervention [22] to improve longer-term health outcomes of stroke survivors, especially when considering evidence that sedentary behaviours are amenable to change [31, 32].

Due to a reciprocal relationship between mood and sedentary behaviour [33], the causal relationship is unclear. Nevertheless, early antidepressant treatment of depressive symptoms after stroke appears to enhance physical recovery from stroke [34], showing that the alleviation of depressive symptoms may lead to less sedentary behaviour. It is therefore plausible that efforts to address depressive symptoms among stroke inpatients may in turn decrease sedentary behaviour. Existing strategies to treat depressive disorders include medication, cognitive behavioural therapy, meditation, and improving health behaviours such as exercise [35, 36]. The use of technology, such as virtual reality (VR), however, to address depressive symptoms is just beginning to receive research and clinical attention.

Virtual reality is defined as a computer-generated digital environment, which the user is immersed in through a head-mounted display (HMD), that can be interacted with as if that environment were real [37]. Presence in VR using a HMD is perceived as higher than in 3D or 2D programs [38]. Virtual reality has garnered substantial attention as a cost-effective treatment approach in stroke rehabilitation [39–41], particularly as a means to supplement existing therapy [42]. For example, VR-based rehabilitation among people with stroke has led to improvements in physical and functional rehabilitation outcomes, including walking speed, balance, and mobility [42, 43], as well as upper limb function [39].

More recently, study results have shown that immersion might be an important aspect of VR to improve mood, especially when no therapeutic elements are incorporated in the program [44]. In fact, immersive HMD VR interventions have been shown to successfully and significantly improve mood among hospital inpatients [45]. Despite evidence that VR is emerging as a method to address the issue with depression [46, 47], the use of VR to improve depressive symptoms among inpatient stroke survivors has yet to be studied. The objective of this paper is to report on the study protocol that is being used to evaluate the feasibility (e.g., recruitment and retention, administrative and participant burden) of a VR entertainment program to improve depressive symptoms in inpatient stroke survivors; and develop an understanding of the influence of VR on depressive symptoms and sedentary behaviour among inpatient stroke survivors.

Our primary hypothesis is that the study protocol will demonstrate sufficient feasibility to support a subsequent larger randomized controlled trial (RCT). We will also examine the effect of the VR program on our primary clinical outcome of depressive symptoms, as well as on secondary outcomes of sedentary behaviour, anxiety, stress, happiness, and motivation to engage in rehabilitation among stroke survivors receiving inpatient rehabilitation.

## Methods

The reporting of this protocol follows the Standard Protocol Items: Recommendations for Intervention Trials guidelines [48]. The description of the intervention follows the CONSORT extension for randomized pilot and feasibility trials [49, 50].

### Trial design

**Fig. 1:**
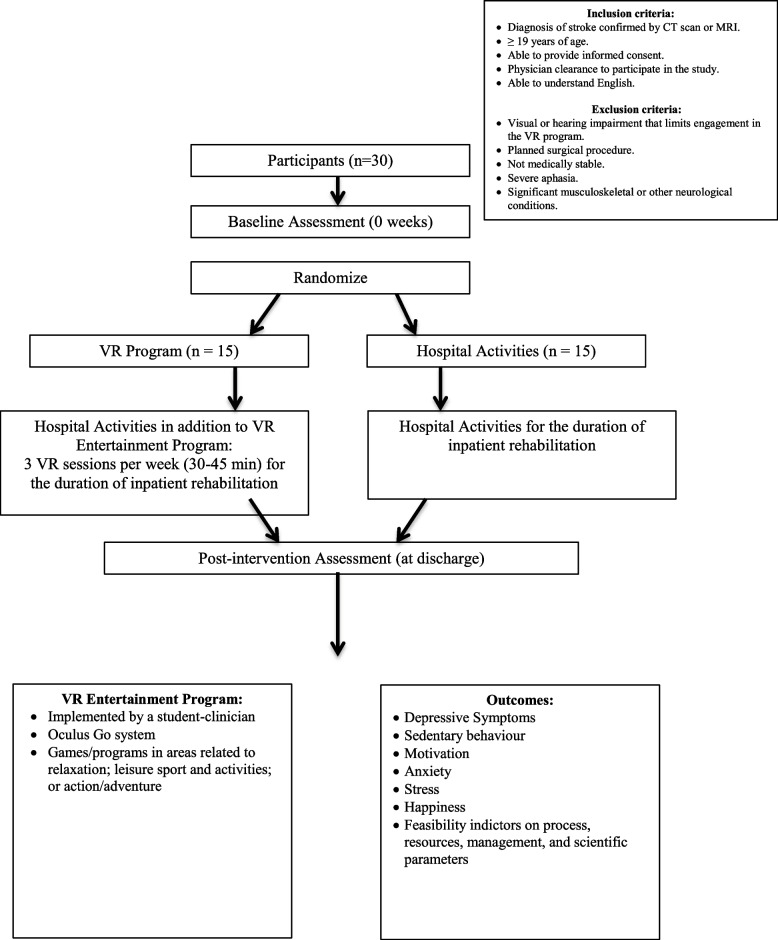
Overview of trial procedures

This pilot study will use a parallel group, single-blinded (tester), randomized controlled trial study design. Figure 1 presents an overview of trial procedures.

### Patient population and sample size

A total of 30 volunteer participants who are receiving inpatient stroke rehabilitation with an expected length of stay of at least 14 days in the rehabilitation unit at Kelowna General Hospital will be recruited. A sample size of 30 is recommended for robust feasibility data used to inform larger trials [51]. Individuals will be included in the study if they have a diagnosis of stroke confirmed by CT scan or MRI; 19 years of age or older; able to provide informed consent; physician clearance to participate in the study (including consideration of cognitive ability, mood (e.g., clinical depression), and functional ability); able to understand English. Individuals will be excluded if they have a visual or hearing impairment that limits engagement in the VR entertainment program; planned surgical procedure, not medically stable, severe aphasia, or have significant musculoskeletal or other neurological conditions.

Enrolled participants will meet with a trained and blinded study assessor who will administer each of the outcome measures, at two timepoints: (i) baseline (T1) and (ii) post-intervention (T2). Each participant will meet with the same study assessor at both time points to ensure consistency in the administration of outcome measures.

### Baseline evaluation (T1)

At baseline, a demographic information questionnaire will be administered. As well, data on medication (e.g., anti-depressives) will be collected, and stroke severity and comorbid conditions will be assessed using the modified Rankin Scale and Functional Comorbidity Index, respectively [52, 53].

#### Feasibility indicators

Data on process, resources, management, and scientific parameters will be collected throughout the study [54]. *Process indicators* will include recruitment (number of subjects/week) and retention rates (percentage of subjects retained), and perceived benefit of the program. *Resource indicators* will include treatment fidelity (number of sessions participated in) and adherence to the VR entertainment program, participant, and tester burden (time to complete assessments), as well as trainer burden (time to administer VR entertainment program). *Management indicators* include equipment challenges (VR downtime due to technical difficulties), and processing time (time from initial contact to enrolment). *Scientific indicators* will include reporting of adverse events due to the VR entertainment program, as well as statistical analysis (see below) of the treatment response on clinical outcomes.

#### Primary clinical outcome

Depressive symptoms will be assessed using the depression subscale from the Hospital Anxiety and Depression Scale (HADS) [55]. Items on the 7-item depression subscale are rated on a four-point (0–3) response scale with higher scores indicating more depressive symptoms [55]. The depression subscale has established excellent internal consistency [56, 57].

#### Secondary clinical outcomes

Sedentary behaviour will be assessed using the patient-reported Measure of Older Adults’ Sedentary Time, with seven sedentary items for patients to report hours and minutes on [58]. The seven sedentary items are watching television or videos/DVDs; using the computer/Internet; reading; socializing with friends or family; driving or riding in a car, or time on public transport; doing hobbies, e.g., craft, crosswords; and doing any other activities.

Motivation to engage in rehabilitation will be assessed using the Situational Motivational Scale (SIMS). The SIMS has been previously used in stroke survivors and consists of 16 items, 4 items per 4 subscales (intrinsic motivation, identified regulation, external; regulation, amotivation), which are scored on a 7-point Likert scale (1 – not at all in agreement to 7 – completely in agreement). Total scores range from 16 to 112 with higher scores indicating greater motivation [59, 60].

Anxiety, stress, and happiness will be assessed using the 7-item anxiety subscale in the HADS, Perceived Stress Scale, and Subjective Happiness Scale [55], respectively. Each item on the anxiety subscale is assessed on a four-point scale. The answer options vary based on the question (e.g., 0 = not at all/only occasionally to 3 = most of the time/a great deal of the time). Total scores range from 0 to 21 with higher scores indicating abnormal anxiety symptoms [55]. The Perceived Stress Scale is answered with 10 questions on a five-point scale (0 = never, 1 = almost never, 2 = sometimes, 3 = fairly often, 4 = very often). Total scores are obtained by reversing responses (e.g., 0 = 4) to four out of ten positive formulated items and summing up all 10 item responses. Higher scores indicate higher stress [61]. The Subjective Happiness Scale has 4 items and is scored on a seven-point scale ranging from 0 = not a very happy person to 7 = a very happy person. To receive the total score, the last item is reversed coded (e.g., 7 = 1) and the mean obtained from the 4-item responses. Higher scores indicate that they consider themselves a very happy person [62].

### Randomization and interventions

After baseline evaluation, participants will be randomly assigned (1:1) to either: the usual care control group, or the VR experimental group. A statistician not involved with the research study will generate the randomization algorithm and provide the research team with sealed envelopes containing the group allocation for sequential participants. An unblinded research coordinator will open the envelope and advise the participants of which group they have been randomized to and advise them of the next steps.

#### Control group

This group will receive usual care hospital activities provided by the health care practitioners, including physical and occupational therapy, as well as speech language services.

#### Experimental group

This group will receive usual care hospital activities, in addition to access and use of the VR entertainment program. Participants in the VR program will receive (1) a 20-min introductory orientation session to the VR program and (2) three 30-min sessions of VR per week for the duration of their inpatient rehabilitation. The VR program will be implemented one-on-one, face-to-face by a member of the research team using the commercially available Oculus Go system developed by Facebook Technologies.

At the time of VR sessions, a hospital staff employee will take the participant to the room where the VR will be administered. At this time, participants will select games/programs in areas related to relaxation (e.g., meditation, travel, and tourism, exploring underwater ocean life); leisure sport and activities (e.g., painting, fishing, mini golf); or action/adventure (e.g., table tennis, duck hunt, racing game). The menu of games/programs was selected by the research team comprised of hospital clinicians (i.e., nurse, social worker, physical therapists), and based on their experiences providing care to stroke survivors and learning about their interests and activities.

After participants select a game/program, the interventionalist will explain the program, including putting on the VR goggles; how to control the game using the handheld remote, as well as game/program instruction. Participants will be instructed that they may stop using the VR at any time and select another game/program or take a break. The participants will never be left alone while playing a game. As well, they will be informed that they may invite friends/family to watch while they are using the VR entertainment program.

We have developed a cleaning and sanitization protocol with the hospital’s Department of Infection Prevention and Control, as follows. Over the course of the study, each participant will have a dedicated Oculus Go system. That is, there will be no sharing of the same system to multiple participants at the same time. After each use, the systems will be cleaned and sanitized, as per the manufacturer’s instructions. Once the patient has completed their participation in the study, the system will be cleaned and sanitized and then placed into a new clean sealable plastic bag. The bag will be sealed and dated. No other participant will be assigned that system for 1 week.

### Follow-up evaluation (T2)

One week prior to the anticipated discharge, the participants will be scheduled for the post intervention (T2) evaluation. All the primary and secondary outcomes collected at T1 will be collected at T2. Participants in the VR group will complete an exit survey at T2 to evaluate their experiences with the VR entertainment program.

### Statistical analyses

Study analyses will consider study feasibility indicators and clinical (statistical) outcomes. Means and standard deviations (for continuous variables) and frequencies and proportions (for categorical variables) will be used to summarize the variables.

#### Feasibility outcomes

Study-specific feasibility criteria have been developed related to process, resources, management, and scientific assessment (Table 1). Feasibility indicators will be dichotomized into either ‘success’ if they meet the a priori criteria, or ‘revise’ if they do not meet the criteria. ‘Success’ will indicate that the protocol is sufficient to move forward with a larger RCT with only small or no adaptation required, and ‘revise’ indicate a need for changes before proceeding.

**Table 1:** Feasibility indicators

Feasibility	Indicator	Criteria for success
**Process**		
Recruitment rate	# of subjects/week recruited	Mean 1 subject/week
Retention rate	% of subjects with retention data	Complete retention > 80% of subjects
Perceived benefit	Satisfaction survey	> 85% will be agree or strongly agree
**Resources**		
Treatment fidelity and adherence	Participate in all VR sessions	> 85% of subjects
Subject and tester burden	Assessments	> 85% of subjects complete in ≤ 60 min
Trainer burden	Time spent administering the program	Mean VR session is 30 min
**Management**		
Equipment	Downtime due to technical issues of VR equipment	> 85% of subjects without any downtime
Processing time	Time from initial contact to enrolment	Mean time is < 5 days
**Scientific**		
Safety	Adverse events from the program activities/advice, or assessments	No major injuries or adverse events reported
Treatment response	ANCOVA comparison between groups	Significant group difference in primary clinical outcome
Treatment effect	Estimate of effect size and variance for future sample size calculations	Data on all relevant variables

#### Clinical outcomes

Intervention effects post-intervention (T2) for the primary and secondary clinical outcomes will be estimated using linear regression including baseline (T1) controlling for age and sex. All analyses will be intention-to-treat. Missing data will be assessed and analysed as appropriate. Analyses will be performed using R software (R Foundation, Vienna) with a significance at 0.05.

## Discussion

The results of this study will increase our understanding about VR, depressive symptoms and sedentary behaviour among individuals receiving inpatient stroke rehabilitation. The information gathered may be used to benefit future patients. Furthermore, if the results are as hypothesized, the protocol will demonstrate sufficient feasibility to support a subsequent larger RCT to examine the effect of VR on depressive symptoms and sedentary behaviour among inpatient stroke survivors. Also, this study will provide insight into how decreased sedentary behaviour during inpatient rehabilitation will affect stroke patient’s sedentary behaviour post inpatient rehabilitation. With depression being a prominent issue in stroke survivors, VR could potentially provide another method to increase activity for stroke survivors.

## Supplementary Information

**Additional file 1.** CONSORT 2010 Flow Diagram.

## Data Availability

Data and study materials will be made available upon reasonable request to the corresponding author.

## Abbreviations

PSD: Post-stroke depression
VR: Virtual reality
HMD: Head-mounted display
RCT: Randomized controlled trial
