# Supplementary material for: The influence of a virtual reality entertainment program on depressive symptoms and sedentary behaviour in inpatient stroke survivors: a research protocol for a pilot randomized controlled trial
Source: Pilot Feasibility Stud. 2022 Oct 22;8:230. doi: 10.1186/s40814-022-01189-8 (PMC9587642; doi:10.1186/s40814-022-01189-8)
Supplement: Supplementary file 1 — Additional file 1. CONSORT 2010 Flow Diagram. [file 40814_2022_1189_MOESM1_ESM.doc]

**CONSORT 2010 Flow Diagram**

**Allocation**

**Analysis**

**Follow-Up**

**Enrollment**

Assessed for eligibility (n= )

Excluded (n= )

♦  Not meeting inclusion criteria (n= )

♦  Declined to participate (n= )

♦  Other reasons (n= )

Analysed (n= )
♦ Excluded from analysis (give reasons) (n= )

Lost to follow-up (give reasons) (n= )

Discontinued intervention (give reasons) (n= )

Allocated to intervention (n= )

♦ Received allocated intervention (n= )

♦ Did not receive allocated intervention (give reasons) (n= )

Lost to follow-up (give reasons) (n= )

Discontinued intervention (give reasons) (n= )

Allocated to intervention (n= )

♦ Received allocated intervention (n= )

♦ Did not receive allocated intervention (give reasons) (n= )

Analysed (n= )
♦ Excluded from analysis (give reasons) (n= )

Randomized (n= )
